# The effects of intermittent escitalopram treatment on impulsivity and inattention in women with premenstrual irritability and anger

**DOI:** 10.1017/S0033291725102055

**Published:** 2025-10-07

**Authors:** Maria Gröndal, Christin Englund, Jakob Näslund, Karl Ask, Elias Eriksson, Stefan Winblad

**Affiliations:** 1 Department of Psychology, University of Gothenburg, Gothenburg, Sweden; 2 Department of Pharmacology, Institute of Neuroscience and Physiology, Sahlgrenska Academy, University of Gothenburg, Gothenburg, Sweden

**Keywords:** Conners Continuous Performance Test, 3rd edition, escitalopram, impulsivity, premenstrual dysphoric disorder, premenstrual irritability and anger, selective serotonin reuptake inhibitors, sensation seeking, urgency, UPPS Impulsive Behavior Scale

## Abstract

**Background:**

Women diagnosed with premenstrual dysphoric disorder (PMDD) report significant symptom relief when treated with selective serotonin reuptake inhibitors, but few studies have addressed the possibility of capturing this effect in behavioral, laboratory-based tests. This study examined the effects of intermittent treatment with escitalopram (vs. placebo) on a behavioral measure of impulsivity and inattentiveness in women reporting high levels of premenstrual irritability and anger.

**Methods:**

Participants (*N* = 27) rated cardinal PMDD mood symptoms over three menstrual cycles using Visual Analogue Scales. In Cycles 2 and 3, participants displaying cyclicity with respect to the irritability/anger item received escitalopram (20 mg) or placebo in a randomized, single-blind, crossover design. The participants completed the Conners Continuous Performance Test (CPT 3) in the luteal phase of the intervention cycles. Additionally, they filled out the UPPS Impulsive Behavior Scale, once in the luteal phase and once in the follicular phase of the placebo cycle.

**Results:**

In line with previous reports, escitalopram caused a significant reduction in self-rated irritability and anger in the luteal phase. When on escitalopram, the participants demonstrated a lower frequency of anticipatory responses and greater consistency in response speed in the CPT 3. With respect to self-reported impulsivity, participants reported higher levels of urgency and lower levels of sensation seeking in the luteal placebo phase versus the follicular phase.

**Conclusions:**

The finding that escitalopram impacted the outcome of the CPT 3 test in women with premenstrual irritability highlights the possible role of impulsivity in this condition.

## Introduction

The distress experienced by a subset of women in the days or weeks preceding menstruation has been studied for many decades (Frank, 1931). Considerable evidence supports the idea that such periodically expressed symptomatology is linked to the fluctuations of sex hormones and their metabolites throughout the menstrual cycle (Hantsoo & Epperson, 2015). Premenstrual complaints encompass both physical and psychological symptoms, but mood-related symptoms, such as irritability and mood swings, are identified as exerting the most substantial harmful impact and functional impairment in the affected individual (Pearlstein, 2016; Studer et al., 2023). Those who experience multiple severe premenstrual complaints may be diagnosed with the mood disorder premenstrual dysphoric disorder (PMDD; American Psychiatric Association, 2013). PMDD is distinguished by the recurring appearance of a minimum of five symptoms during the luteal phase with full remission of symptoms in the week after the onset of menses. While the existence of premenstrual mood symptoms is widely documented, their relationships with transdiagnostic behavioral factors, such as impulsivity and inattention, are less well understood.

It is well-established that premenstrual symptoms can be effectively reduced by treatment with selective serotonin reuptake inhibitors (SSRIs; Eriksson et al., 1995; Sundblad et al., 1992). The symptom reduction occurs rapidly within a few days after commencing SSRI medication, and the treatment can therefore be administered intermittently, typically from ovulation until the onset of menstruation (Eriksson et al., 2008; Steinberg et al., 2012). Importantly, the premenstrual symptoms particularly alleviated by intermittent SSRI treatment are those with emotional characteristics, specifically irritability/anger and affect lability (Landén et al., 2009).

The effectiveness of SSRIs in mood-related premenstrual complaints suggests serotonergic transmission to play a role in the pathophysiology of these conditions (Yonkers et al., 2008). In line with this, sex hormones and their associated receptors are known to modulate serotonin transmission (Rubinow et al., 1998) and are found abundantly in brain regions responsible for regulating emotions and their behavioral outcomes, such as the amygdala, hippocampus, and prefrontal cortex (Brinton et al., 2008; Hara et al., 2015). Support for an involvement of serotonin in PMDD includes findings that tryptophan depletion – a precursor to serotonin – can induce premenstrual mood symptoms (Menkes et al., 1994), while serotonin receptor antagonists may block the symptom-relieving effects of SSRIs (Roca et al., 2002). Moreover, a positron emission tomography study has recently provided direct support for an altered serotonergic function during the premenstrual phase in PMDD patients (Sacher et al., 2023).

Impulsivity-related disabilities and associated cognitive complaints often occur alongside dysfunctional affective symptoms in psychiatric conditions (Okon-Singer et al., 2015). As serotonin is suggested to play an important physiological role in the regulation of impulsivity (da Cunha-Bang & Knudsen, 2021; Dalley & Roiser, 2012), it is of interest to assess whether enhanced impulsivity may be a facet of premenstrual dysphoria, and, if so, whether SSRI treatment may impact this symptom. In line with this, previous research has found increases in impulsive, risky, or maladaptive behaviors during the luteal phase (Eisenlohr-Moul et al., 2022), as well as positive correlations between emotion-related impulsivity and the severity of other PMDD symptoms (Dawson et al., 2018). While impulsivity has been suggested as a trait feature in patients diagnosed with PMDD (Yen et al., 2011), periodically exacerbated impulsivity during the luteal phase has also been observed (Ko et al., 2014). When examining different types of impulsivity, Petersen et al (2016) found behavioral but not cognitive trait impulsivity to be elevated in PMDD patients (vs. controls). In sum, previous findings highlight the importance of studying different varieties of impulsivity.

The purpose of this study, aiming to shed further light on the possible cognitive components of impulsivity in premenstrual irritability, was two-pronged. First, women with premenstrual irritability were asked to complete the UPPS Impulsive Behavior Scale (UPPS; Whiteside et al., 2005) once in the follicular phase and once in the luteal phase of the placebo cycle, to capture possible phase-related changes in self-rated impulsivity. Second, the same participants were subjected to the Conners Continuous Performance Test (CPT 3), a behavioral task that captures attention-related and executive processes linked to impulsive behavior, to assess whether administration of an SSRI may impact this measure. CPT 3 is often referred to as a measure of sustained attention, but it has also been argued that it should instead be regarded as multidimensional, reflecting the integrated performance of perceptual, cognitive, and motor functions (Scimeca et al., 2021; van den Bosch et al., 1996). CPT 3 has been extensively used in psychiatric research on attention-related conditions, such as attention-deficit hyperactivity disorder (Huang-Pollock et al., 2012) and schizophrenia (Nieuwenstein et al., 2001), but has also been used to evaluate the effects of SSRI treatment in patients with depression (Hart et al., 1998; Koetsier et al., 2002). Given the suggested impact of serotonin on impulsivity (Roberts et al., 2020), as well as the possible association between phase-related changes in premenstrual impulsivity and irritability, respectively, we deemed it justified to assess whether an SSRI known to effectively dampen premenstrual irritability may also reduce impulsivity as reflected by the outcome of CPT 3.

## Material and methods

### Study design and settings

The study had a randomized and placebo-controlled crossover design. For logistical reasons, the treatment was single-blind rather than double-blind – that is, the participants but not the staff were unaware of the order of the escitalopram cycle and the placebo cycle, respectively. Participants received 1,000 SEK (~100 USD) for each of the three lab visits and were additionally reimbursed for travel and/or loss of income due to these visits. All procedures were approved by the University of Gothenburg Institutional Review Board (Dnr854–13/EudraCTnr2012–000309-60). In a previous article based on the same study, the outcome of tests reflecting aggressive behavioral responses was reported (Gröndal et al., 2025).

### Participants

Participants were recruited using advertisements in social media and a local newspaper seeking women with premenstrual dysphoria. Interested individuals were directed to a phone screening with a research nurse. Those who reported having regular menstrual cycles and symptoms of irritability and anger during the luteal phase, without ongoing medical or hormonal treatment, were directed to a screening visit with a medical doctor.

Exclusion criteria were as follows: psychiatric illness within <1 year (excl. PMDD), moderate or high risk of suicide, ongoing psychotropic medication (including anxiolytics and sleeping pills), ongoing structured psychotherapy, ongoing medical treatment with hormonal contraceptives, previous negative experience with SSRI treatment, ongoing breastfeeding, ongoing pregnancy or risk of becoming pregnant during the study period, and difficulty understanding the purpose of their participation in the study (e.g. due to language barriers).

Participants who did not meet any exclusion criteria were asked to complete daily symptom ratings for one full menstrual cycle, beginning on the first day of the menstrual period and ending on the first day of the subsequent menstrual period. For inclusion in the study, participants were required to (a) show at least a 50% increase in irritability/anger symptoms from the follicular phase (average of days 6–10) to the luteal phase (average of days −5 to −1) as measured by means of a Visual Analogue Scale (VAS; 1–100 mm) and (b) have a mean rating of irritability/anger ≥30 mm over the last 5 days of the menstrual cycle. Participants who did not exhibit sufficient symptoms during the first menstrual cycle were asked to complete daily symptom ratings for an additional menstrual cycle. If they met the inclusion criteria during the subsequent cycle, they were included in the study. All included participants were requested to maintain daily ratings of symptom severity using the same VAS instrument throughout the remaining three menstrual cycles of the study period.

### Assessment of premenstrual mood symptoms

Using an online questionnaire hosted on the Qualtrics online survey platform (https://www.qualtrics.com), participants reported on a daily basis to what extent they had experienced each of the cardinal mood symptoms of PMDD: *irritability/anger*, *depressed mood*, *mood swings*, and *tension/anxiety* using VAS scales ranging from 0 (*not at all*) to 100 (*maximal*). Participants gained access to the questionnaire through a personal QR code assigned to them during the screening visit. If a participant failed to submit their responses for more than 3 consecutive days, they received a text message reminder.

### Impulsivity measures

#### UPPS Impulsive Behavior Scale

The UPPS Impulsive Behavior Scale (UPPS; Whiteside et al., 2005) consists of four facets of trait impulsivity: urgency, (lack of) premeditation, (lack of) perseverance, and sensation seeking. Participants rated their agreement with a total of 45 statements using a four-point scale (1 = *agree strongly* and 4 = *disagree strongly*). Participants filled out the UPPS once in the follicular phase and once in the luteal phase of the placebo cycle. During the follicular phase, participants were asked to report how they generally behave during periods without premenstrual symptoms. In the luteal phase, participants were asked to report how they generally behave during periods with premenstrual symptoms.

The *urgency* subscale consists of 12 items (e.g. ‘I have trouble controlling my impulses’) and measures the tendency to engage in impulsive behaviors to alleviate negative affect (luteal phase: ordinal *α* = .93, ordinal *ω*_total_ = .93; follicular phase: ordinal *α* = .84, ordinal *ω*_total_ = .83). The *(lack of) premeditation* subscale consists of 11 items (e.g. ‘I am a cautious person’) and measures the tendency not to reflect or deliberate on the consequences of behaviors before engaging in them (luteal phase: ordinal *α* = .85, ordinal *ω*_total_ = .84; follicular phase: ordinal *α* = .67, ordinal *ω*_total_ = .67). The *(lack of) perseverance* subscale consists of 10 items (e.g. ‘I finish what I start’) and measures the inability to remain focused on a difficult or boring task and to resist distractions (luteal phase: ordinal *α* = .79, ordinal *ω*_total_ = .79; follicular phase: ordinal *α* = .79, ordinal *ω*_total_ = .76). Finally, the *sensation seeking* subscale consists of 12 items (e.g. ‘I’ll try anything once’) and measures an individual’s openness to trying risky, exciting activities and tendency to enjoy such activities (luteal phase: ordinal *α* = .75, ordinal *ω*_total_ = .73; follicular phase: ordinal *α* = .81, ordinal *ω*_total_ = .81). For each subscale, a total score was calculated by summing the individual item ratings (after reverse scoring where appropriate), such that higher scores meant higher impulsivity.

#### Conners Continuous Performance Test 3

A computerized version of the CPT 3 (Conners, 2014) was administered in a lab setting at the University of Gothenburg and measured two aspects of attention: impulsivity and inattentiveness. Participants were instructed to respond as fast as possible with the index finger of the dominant hand when any letter, except the letter ‘X’, appeared on the screen. The presentations of letters were separated by an interval of 1, 2, or 4 s with a display time of 250 ms. A total of 360 trials were displayed over 6 blocks (sets of trials). The following CPT 3 parameters were computed: *detectability* (ability to discriminate targets [non-X] from nontargets [X]), *hit reaction time* (HRT; average response speed to target stimuli in ms), *hit reaction time standard deviation* (HRT *SD*; response speed consistency during the entire administration), *omission errors* (failures to respond to target stimuli), *commission errors* (responses to nontarget stimuli), *perseverations* (rate of anticipatory, repetitive, or random responses), and *variability* (response speed consistency between segments of the administration). The administration time for the game was 14 min (excluding time for instructions and practice trials).

The following combination of parameters was regarded as associated with the *inattentiveness* dimension: low detectability, high number of omissions, high/low number of commissions, normal or slow HRT, high/low HRT *SD*, and high/low variability. The following combination of parameters was regarded as associated with the *impulsivity* dimension: a high number of commissions, a high number of perseverations, and a fast HRT.

### Procedure

**Figure 1.**
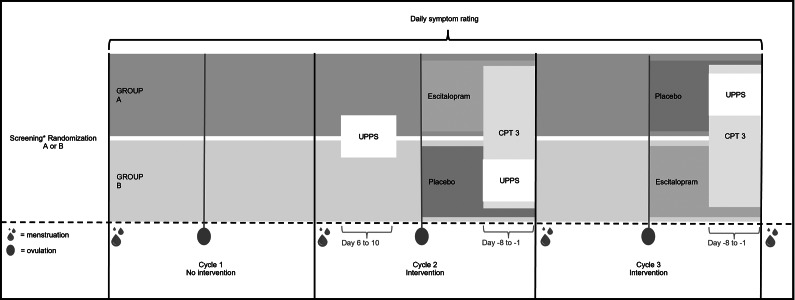
Flowchart of the study procedure.

A flow chart of the study procedure is presented in Figure 1. The study extended over three menstrual cycles: Cycle 1 (without intervention) and Cycles 2 and 3 (with intervention). In Cycle 1, participants recorded emotional symptoms over a complete menstrual cycle to validate the presence of premenstrual irritability/anger in the luteal phase (for details, see Participants). For Cycles 2 and 3, participants were randomly assigned to one of two groups for the treatment phase. One group received active SSRI treatment (escitalopram, 20 mg) in Cycle 2 and placebo in Cycle 3, while the other group received the treatments in the reverse order (i.e. first placebo, then escitalopram). The packaging and tablets were identical in appearance for the escitalopram and placebo treatments, respectively.

Participants completed CPT 3 twice, once in the luteal phase of the escitalopram cycle and once in the luteal phase of the placebo cycle. In addition, they filled out the UPPS twice (once in the follicular phase and once in the luteal phase of the placebo cycle).

In addition to the CPT 3 testing, the participants were also the subject of sampling for an analysis of the cerebrospinal fluid (the results of which will be published elsewhere) in both luteal phases and in one of the follicular phases. It was deemed important that both the order of the cycle phases and the order of the two treatments given in the luteal phase were randomized, and also that the follicular phase sampling did never take place shortly after a luteal phase where the participant had received active drug; hence, follicular sampling occurred in the first cycle of subjects receiving escitalopram in this cycle and in the second cycle of those receiving placebo in the first. This is why a double-blind design was not feasible.

### Outcome measures

The outcome measures for the daily symptom ratings of irritability/anger, depressed mood, mood swings, and tension/anxiety were the average ratings for days −5 to −1 of the placebo and escitalopram menstrual cycles, respectively.

The outcome measures for the self-reported impulsivity were the total score for each of the subscales of UPPS (urgency, [lack of] premeditation, [lack of] perseverance, and sensation seeking) in the luteal and follicular phase. When evaluating the CPT 3 parameters, we used T-scores, which are recommended when determining change scores in an entire group, as the T-value adjusts the scores relative to age (Conners, 2014). All CPT 3 outcome variables were automatically computed using the standardized scoring algorithms implemented in the CPT 3 software (Conners, 2014). Each of the included parameters in CPT 3 was treated as a separate outcome measure.

### Statistical analyses

The alpha level was set at 5% and all computations were performed in R (version 4.1.2). Hedges’ *g* was used as an effect size for mean comparisons, as it corrects for bias in smaller samples (Lakens, 2013).

Changes in daily symptom ratings between the escitalopram and placebo treatments were assessed with dependent *t*-tests.

Dependent *t*-tests with bootstrapped mean differences (bootstrap replicates = 5,000, using the *boot* package version 1.3.31 for R) were used to compare the self-reported subscales in UPPS in the luteal and follicular phases. The bootstrap function was applied because many of the tested variables were not normally distributed.

Results for CPT 3 during the escitalopram and placebo treatments were also compared using dependent *t*-tests with bootstrapped mean differences (bootstrap replicates = 5,000, using the *boot* package version 1.3.31 for R). To control for potential practice and order effects in CPT 3, we conducted linear mixed models with time (practice) and sequence (order) as fixed effects, condition as a covariate, and a random intercept for participants.

## Results

Demographic information and mean scores of the symptom ratings of the participants at baseline (Cycle 1), in the escitalopram cycle, and the placebo cycle, respectively, have been reported earlier (Gröndal et al., 2025) but are nevertheless included for the sake of convenience (Table 1). The participants included in the analyses were tested between 8 and 1 day before the next menstruation.

**Table 1.** Participants’ demographics, self-reported symptoms, and time of testing

	Baseline	Placebo	Escitalopram
Age (year)	32.78 (6.12)	-	-
Educational level			
University degree	85.19%	-	-
No university degree	14.81%	-	-
Employment			
Working part- or full-time	85.19%	-	-
Studying	14.81%	-	-
Daily symptom ratings (days −5 to −1)			
Irritability/anger	56.35 (19.53)	32.49 (19.00)	(22.16)^a^ **
Depressed mood	42.59 (18.23)	21.61 (16.25)	(19.57)^b^
Mood swings	49.21 (23.58)	27.08 (15.78)	10.55 (18.09)^c^ **
Tension/anxiety	56.60 (23.05)	31.10 (19.07)	21.16 (24.95)^d^
Testing day (days before menses)	-	−3.31 (2.01)	−3.46 (1.50)

*Note:* Data previously presented in (Gröndal et al., 2025). Values represent *M* (SD) or percent of the total sample. Changes in symptom ratings were compared between the escitalopram and the placebo cycles.

a Hedges’ *g* = −0.906 [0.307, 1.504].

b Hedges’ *g* = −0.390 [−0.106, 0.886].

c Hedges’ *g* = −0.974 [0.294, 1.654].

d Hedges’ *g* = −0.538 [−0.036, 1.111].

^**^ *p < .01* as indicated by dependent *t-*tests.

A total of 203 individuals completed phone screening, 98 of whom were eligible for medical visits and daily symptom ratings. Of those, 52 reported sufficient symptoms according to the inclusion criteria, 13 of whom dropped out during the study. Out of the remaining 39 participants, 33 completed the UPPS Impulsive Behavior Scale in the follicular and placebo-treated luteal phase, respectively, whereas 28 completed the CPT 3 twice, once during the placebo cycle and once during the escitalopram cycle. Cases of missing values were due to unexpected sick leave in a critical period by a member of the staff conducting these tests. Of the 28 participants who completed both CPT 3 assessments, one was excluded from the analysis due to a 13-day interval between one of the tests and the onset of menstruation. The final CPT 3 sample thus consisted of 27 participants with data from both test occasions.

As previously reported (Gröndal et al., 2025), significant differences in the daily symptom ratings between the escitalopram and placebo cycles were observed in self-reported irritability/anger and mood swings. No significant differences between escitalopram and placebo were observed in depressed mood and tension/anxiety ratings (Table 1).

### UPPS Impulsive Behavior Scale

Participants reported significantly higher levels of urgency in the luteal phase compared with the follicular phase (see Table 2). Furthermore, a significant mean difference was observed for sensation seeking, with lower levels reported in the luteal phase compared with the follicular phase. No significant differences were detected across the cycle phases for (lack of) reflection and (lack of) perseverance.

**Table 2.** Means and standardized mean differences for the UPPS subscales in the follicular and luteal phases (*N* = 24)

Subscale	Follicular	Luteal	*g*	95% CI_*g*_
	*M* (SD)	*M* (SD)		
Urgency	26.88 (5.72)	33.92 (7.17)	1.046*	[0.557, 1.742]
Premeditation (lack)	24.29 (3.94)	26.54 (5.61)	0.447	[−0.067, 1.002]
Perseverance (lack)	22.88 (4.79)	25.04 (4.41)	0.453	[−0.112, 1.689]
Sensation seeking	28.83 (6.52)	22.83 (5.46)	−0.847*	[−1.345, −0.407]

*Note*: Higher scores mean higher impulsivity. *g* = Hedges’ *g.* * = 95% CI_g_ does not include 0.

### Conners Continuous Performance Test 3

The parameters perseverations and HRT *SD* in CPT 3 were significantly higher during the placebo cycle than during the escitalopram cycle (see Table 3). These results indicate that participants had a lower rate of anticipatory responses, had a higher ability to maintain attention during the task, and processed stimuli more efficiently during the active treatment (vs. placebo) cycle. No differences were observed between the escitalopram cycle and the placebo cycle with respect to the other CPT 3 parameters.

**Table 3.** Comparison of CPT 3 parameters between the placebo and escitalopram cycles (*N* = 27)

CPT 3 parameter	Placebo	Escitalopram	*g*	95% CI_*g*_
	*M* (SD)	*M* (SD)		
Detectability	49.63 (8.37)	47.07 (8.89)	−0.286	[−0.732, 0.123]
Omissions	48.33 (5.05)	48.11 (4.39)	−0.045	[−0.480, 0.419]
Commissions	50.15 (8.89)	47.11 (8.59)	−0.336	[−0.712, 0.005]
Perseverations	49.59 (6.16)	47.81 (2.42)	−0.367*	[−0.667, −0.128]
HRT	47.19 (6.76)	48.41 (7.83)	0.162	[−0.103, 0.413]
HRT *SD*	44.04 (7.08)	41.48 (6.34)	−0.368*	[−0.796, −0.028]
Variability	47.93 (8.06)	45.41 (6.96)	−0.324	[−0.803, 0.145]

*Note*: HRT, hit reaction time; HRT *SD*, hit reaction time standard deviation; *g*, Hedges’ *g.* * = 95% CI_g_ does not include 0.

To estimate practice and sequence effects on CPT 3 parameters, we fitted linear mixed models with participants included as a random factor. The analyses indicated minimal and nonsignificant changes over time (practice effects) and no clear sequence effects on CPT 3 performance. Results are provided in the Supplementary Materials.

## Discussion

The self-report measure of impulsivity, the UPPS Impulsive Behavior Scale, suggested higher levels of urgency and lower levels of sensation seeking in the luteal phase than in the follicular phase. As reported previously (Gröndal et al., 2025), and in line with previous studies, participants reported a significant decrease in irritability/anger and mood swings in the daily symptom ratings in the luteal phase of the escitalopram cycle compared with the placebo cycle. Regarding the laboratory measures of attention and impulsivity, participants in the luteal phase had a lower rate of anticipatory responses and higher response speed consistency in the escitalopram cycle than in the placebo cycle.

With respect to self-reported impulsivity, the finding that urgency increased significantly among participants in the luteal phase compared with the follicular phase suggests emotion-related impulsivity in premenstrual complaints and aligns with previous findings (Dawson et al., 2018). In contrast to urgency, participants’ sensation seeking was found to decrease in the symptomatic phase. Unlike the other facets of the UPPS, sensation seeking has been associated with adaptive functioning (Ravert et al., 2013). For instance, it is associated with extraversion (Whiteside & Lynam, 2001), which, in turn, is associated with well-being (Costa & McCrae, 1992). Future studies on the role of various forms of impulsivity in PMDD appear warranted.

Heightened impulsivity has been suggested to become exacerbated in the luteal phase (Ko et al., 2014) and to be a trait feature of patients with PMDD (Yen et al., 2011). The current study did not investigate the trait dimension of impulsivity. That is, we do not know how participants’ self-rated levels of impulsivity would compare with a population without premenstrual irritability and anger. Instead, our study shows how different types of impulsivity vary within individuals between symptomatic and nonsymptomatic phases.

The parameters of the impulsivity dimension of CPT 3 are efficient in identifying rapid-response impulsivity and failures to suppress inappropriate actions in situations where the individual is required to make time-limited evaluations and discriminations. The current findings suggest that escitalopram improved the ability of the participants to inhibit anticipatory responses, which is in line with previous studies of how serotonin may impact impulsive action or behavioral inhibition (Miyazaki et al., 2012; Roberts et al., 2020). Moreover, participants also processed stimuli more effectively throughout the task compared with the placebo cycle, indicating improved attention during the active treatment cycle. Attention performance has previously been studied, albeit to a limited extent, in different cycle phases in women with PMDD (Le et al., 2020), but there is currently no consensus on how attention-related performance is affected in women with severe premenstrual complaints. To the best of our knowledge, this is the first report addressing the possible impact of an SSRI on the outcome of the CPT 3.

The above findings need to be interpreted with caution, as CPT 3 includes several parameters in the impulsivity and inattentiveness dimensions, and only one parameter in each dimension was significantly improved by escitalopram treatment. Thus, we need to limit our claim such that some aspects of the multidimensional impulsivity and inattention constructs appear to improve as a result of SSRI medication. Caution also needs to be applied when linking the CPT 3 findings to the reduction of self-reported irritability/anger and mood swings in the escitalopram (vs. placebo) cycle. While the experience of negative emotions can be exacerbated in the presence of dysfunctional impulsivity (Barratt & Slaughter, 1998; Lynam & Miller, 2004) and inattention (Hammar et al., 2022), the current findings do not reveal whether reduced impulsivity and inattention actively contribute to the reduced symptom ratings. Laboratory research on premenstrual disorders is in its infancy, and it remains unclear whether the associated cognitive impairments can be attributed to negative affective experiences and psychological symptoms or whether they are direct effects of hormonal dysregulation or sensitivity. Nonetheless, the current findings indicate that effects on impulsivity and inattention may be important components in understanding the therapeutic effects of SSRIs in premenstrual irritability/anger and mood swings.

Some limitations of the current study should be acknowledged. First, similar to most laboratory-based studies of premenstrual complaints and PMDD, our study is based on a small sample of participants (Le et al., 2020). Although statistical power is improved in the present study due to its crossover design, the small sample size may have prevented the detection of small but potentially important associations. Second, since the tested hypothesis was that there may be a link between enhanced impulsivity and premenstrual irritability (rather than other premenstrual complaints), for inclusion we required patients to display cyclicity with respect to this particular PMDD symtom in the Diagnostic and Statistical Manual of Mental Disorders, fifth edition - but no other symptoms; moreover, for logistical reasons, the inclusion of participants was based solely on symptom ratings from a single menstrual cycle. In contrast, the current diagnostic criteria for PMDD require the presence of at least five symptoms (American Psychiatric Association, 2013), the cyclicity of which should be confirmed during two consecutive cycles. Thus, it would be inaccurate to generalize the current findings to individuals diagnosed with PMDD. Third, most participants in the current study exhibited a high level of education, and the sample consisted entirely of students and individuals with employment. Consequently, our sample represents a cohort of high-functioning women. Fourth, for logistical reasons, the staff was not blinded to treatment allocation, which possibly might have impacted the evaluation of the effect of escitalopram on the subjectively rated symptom severity, though to a lesser extent than if this assessment had been undertaken by the investigators rather than – as was the case – by the participants. We deemed the single-blind design justified, given that the primary aim of this trial was not to address the well-established efficacy of an SSRI on premenstrual irritability but the possible effect of the drug on an objective (or semi-objective) measure – that is, the outcome of the CPT 3. Finally, it should be emphasized that this was an exploratory, hypothesis-generating study, the results of which should be interpreted with caution until replicated.

In conclusion, the current study showed that intermittent treatment with escitalopram during the symptomatic luteal phase of the menstrual cycle may have a beneficial impact on specific aspects of impulsivity and inattentiveness in women with severe premenstrual irritability and anger. Moreover, our findings showed that self-reported urgency increased, whereas self-reported sensation seeking decreased in the luteal versus the follicular phase, suggesting that different facets of impulsivity may be of different significance to premenstrual complaints. The current study advances the understanding of the roles of impulsivity and inattentiveness, both in terms of the therapeutic action of SSRIs and in terms of natural fluctuations across the menstrual cycle.

## Supporting information

Gröndal et al. supplementary material Gröndal et al. supplementary material

## Supplementary material

The supplementary material for this article can be found at http://doi.org/10.1017/S0033291725102055.
