# Supplementary material for: The effects of intermittent escitalopram treatment on impulsivity and inattention in women with premenstrual irritability and anger
Source: Psychol Med. 2025 Oct 7;55:e301. doi: 10.1017/S0033291725102055 (PMC12527498; doi:10.1017/S0033291725102055)
Supplement: Gröndal et al. supplementary material [file S0033291725102055sup001.docx]

Supplementary Materials

**Table S1**

*Practice and Sequence Effects in CPT 3 Performance*

| CPT 3 parameter | Session 1  Placebo first | Session 2  Placebo first | Session 1  Escitalopram first | Session 2  Escitalopram first | Practice effect  Session 1 to 2 | Sequence effect  (treatment order) |
| --- | --- | --- | --- | --- | --- | --- |
| Detectability | 50.29 ± 9.02 | 44.36 ± 7.98 | 50.00 ± 9.19 | 48.92 ± 7.91 | -3.50 ± 1.79 (t = -1.96) | 2.14 ± 2.76 (t = 0.77) |
| Omissions | 49.50 ± 6.07 | 47.29 ± 2.89 | 49.00 ± 5.57 | 47.08 ± 3.48 | -2.07 ± 1.05 (t = -1.98) | -0.35 ± 1.48 (t = -0.24) |
| Commissions | 49.86 ± 8.64 | 44.14 ± 6.96 | 50.31 ± 9.28 | 50.46 ± 9.50 | -2.78 ± 1.50 (t = -1.85) | 3.38 ± 2.96 (t = 1.14) |
| Perseverations | 47.79 ± 1.76 | 47.43 ± 0.94 | 48.23 ± 3.37 | 51.54 ± 8.42 | 1.48 ± 0.86 (t = 1.71) | 2.28 ± 1.53 (t = 1.49) |
| HRT | 48.21 ± 7.44 | 49.79 ± 9.23 | 46.92 ± 6.01 | 46.08 ± 6.03 | 0.36 ± 1.07 (t = 0.34) | -2.50 ± 2.62 (t = -0.95) |
| HRT *SD* | 44.07 ± 7.72 | 39.21 ± 4.48 | 43.92 ± 7.27 | 44.00 ± 6.63 | -2.39 ± 1.23 (t = -1.95) | 2.32 ± 2.24 (t = 1.04) |
| Variability | 47.93 ± 9.61 | 43.14 ± 3.30 | 47.85 ± 8.98 | 47.92 ± 6.37 | -2.35 ± 1.90 (t = -1.24) | 2.35 ± 2.17 (t = 1.08) |

*Note*. HRT = Hit reaction time. HRT *SD* = Hit reaction time standard deviation.
